# Evinacumab Treatment of Homozygous Familial Hypercholesterolemia Patients in a Real-World Setting: An Overview After the First Year of Therapy

**DOI:** 10.1016/j.aed.2026.03.004

**Published:** 2026-03-25

**Authors:** Laura Volpi, Dominik Spira, Knut Mai, Ilja Demuth, Elisabeth Steinhagen-Thiessen, Ursula Kassner, Thomas Bobbert

**Affiliations:** 1 Charité – Universitaetsmedizin Berlin, Corporate Member of Freie Universität Berlin and Humboldt-Universität zu Berlin, Department of Endocrinology and Metabolism, European Reference Network on Rare Endocrine Diseases (ENDO-ERN), Berlin, Germany; 2 German Center for Diabetes Research (DZD), München-Neuherberg, Germany; 3 DZHK (German Centre for Cardiovascular Research), Partner Site Berlin, Berlin, Germany; 4 Department of Human Nutrition, German Institute of Human Nutrition Potsdam-Rehbruecke, Nuthetal, Germany; 5 Charité - Universitätsmedizin Berlin, BCRT - Berlin Institute of Health Center for Regenerative Therapies, Berlin, Germany

**Keywords:** evinacumab, homozygous familial hypercholesterolemia, angiopoietin-like 3 protein inhibitors

## Abstract

**Background and Objectives:**

Homozygous familial hypercholesterolemia (HoFH) is a genetic disorder characterized by lifelong low-density lipoprotein cholesterol (LDL-C) elevation, which results in early onset of atherosclerotic cardiovascular disease. Recently, evinacumab, an angiopoietin-like 3 protein inhibitor, became available to treat patients affected by HoFH. We aimed to investigate safety and efficacy data in HoFH in clinical practice.

**Methods:**

Our HoFH cohort (*n* = 7) included 4 patients with 2 LDL receptor (*LDLR)*-related or *LDLRAP1*-related copies of the identical variant and 3 patients with 1 copy each of 2 different *LDLR*-related variants. All subjects were receiving maximally tolerated lipid-lowering therapy, 4 patients had additionally lipoprotein apheresis. The average LDL-C at baseline was 222 mg/dl. Each patient was treated with evinacumab at a dose of 15 mg/kg intravenously every 4 weeks, except for one patient, who received it every 6 to 8 weeks. In one patient, therapy was discontinued after 3 doses, due to a suspected allergic reaction. Clinical and lipid parameters were regularly evaluated over 52-weeks.

**Results:**

An average decrease from baseline in LDL-C of −68.9 ± 14.0% (*P* < 0,001) was observed throughout the treatment period of 52 weeks. In one patient, reductions of 56% and 52% were achieved after therapy intervals of 6 and 8 weeks, respectively. Five patients reached their LDL-C target, lipoprotein apheresis could be interrupted in 2 patients due to high efficacy of evinacumab. No side effects were observed, except for a suspected allergic reaction.

**Conclusion:**

Evinacumab on top of the best standard of care treatment is an effective treatment to further reduce LDL-C levels in HoFH patients.

**Highlights**

• Homozygous familial hypercholesterolemia (HoFH) is a rare genetic disorder causing lifelong low-density lipoprotein-cholesterol (LDL-C) elevation and premature atherosclerotic cardiovascular disease
• Conventional lipid lowering therapies are often insufficient in HoFH patients, highlighting the need for novel approaches to achieve LDL-C targets
• In our cohort, an average decrease from baseline in LDL-C of −68.9 ± 14.0% was observed throughout the entire treatment period of 52 weeks
• Evinacumab promises to bring a transformation in the therapeutic paradigm for patients affected by HoFH, who, despite requiring life-long therapy, will benefit from a less invasive treatment with a different impact on their daily lives

**Clinical Relevance**

This study evaluates real-world safety and efficacy of evinacumab in homozygous familial hypercholesterolemia, addressing the limited understanding of its long-term effects and providing important insights into outcomes and treatment impact in this high-risk population.

## Introduction

Homozygous familial hypercholesterolemia (HoFH) is a rare autosomal semi-dominant disorder characterized by lifelong, therapy-resistant elevation of serum low-density lipoprotein cholesterol (LDL-C). This is associated with a substantial increased risk to develop early onset of atherosclerotic cardiovascular disease and premature death.^1^, ^2^, ^3^

HoFH is caused by pathogenic variants in genes responsible for LDL-C clearance, including those coding for the LDL receptor (*LDLR*), apolipoprotein B, and proprotein convertase subtilisin/kexin type 9 (*PCSK9*). These variants result in reduced or even absent hepatic uptake of LDL-C, leading to elevated levels in circulation.^4^^,^^5^

Current treatments for HoFH primarily aim to lower LDL-C, but therapeutics like statins, ezetimibe, bempedoic acid, and PCSK9 inhibitors target the LDLR pathway.^6^^,^^7^ In HoFH patients, the effectiveness of these compounds is limited due to the impaired or absent function of *LDLRs*, which is essential for their mechanism of action.^8^, ^9^, ^10^

Alternative therapies such as lipid apheresis and lomitapide offer more effective LDL-C reduction for those patients, yet have notable limitations under different aspects.^11^ Lipid apheresis is an invasive procedure that requires frequent sessions (once or twice per week), imposing a significant burden on patients' quality of life. Furthermore, it is not entirely effective, as the LDL reduction is only temporary and fluctuating, showing the typical zigzag pattern with the LDL-C rapidly increasing after each session.

By contrast, lomitapide, an oral medication that inhibits lipoprotein production, can significantly reduce LDL-C levels. However, its use is often restricted due to gastrointestinal side effects and the potential for elevated liver enzymes.^10^^,^^12^, ^13^, ^14^

Since no single therapy fully addresses the needs of HoFH patients, a combination of treatments is required.^15^ The introduction of evinacumab, a treatment that functions independently of *LDLRs*, has proven especially beneficial for patients unresponsive or only partly responsive to conventional therapies.

Evinacumab is a fully human monoclonal antibody that targets and inhibits angiopoietin-like 3 (ANGPTL3), a protein involved in lipid metabolism through the inhibition of enzymes like lipoprotein lipase and endothelial lipase (EL). These enzymes are endogenous lipases, which catalyze the hydrolysis of the triglycerides (TGs) and of the very low-density lipoprotein in serum. In some studies, it has been observed that a loss of function of the gene encoding ANGPTL3 is associated with low levels of TGs, but also with lower levels of LDL-C and high-density lipoprotein cholesterol (HDL-C).^16^, ^17^, ^18^

By inhibiting ANGPTL3, evinacumab preserves the activity of the enzymes lipoprotein lipase and EL, resulting in lower levels of TGs, LDL-C, and HDL-C.^19^ Recently, EL has been identified as an enzyme involved not only in the catabolism of high-density lipoproteins, but also in the metabolism of apolipoprotein B–containing lipoproteins. Therefore, enhancement of its activity through evinacumab promotes the generation of lipid-poor very low-density lipoprotein remnant particles, thereby accelerating their clearance from the bloodstream. Consequently, the availability of LDL precursors declines, resulting in reduced LDL-C concentrations independently of LDL-receptor activity, making this mechanism particularly relevant for patients with HoFH.^19^, ^20^, ^21^

In the pivotal Phase 3 ELIPSE HoFH clinical trial, evinacumab, compared to placebo, reduced LDL-C levels by 49% from baseline in HoFH patients despite concurrently using other lipid-lowering therapies (LLTs) such as statins and PCSK9 inhibitors. This significant additional reduction was sustained over the treatment period, demonstrating its efficacy in patients who showed insufficient response to conventional therapies.^2^

Other recent clinical trials have demonstrated effective reduction of LDL-C in HoFH patients, alongside a well-tolerated safety profile.^14^^,^^22^^,^^23^

To summarize, evinacumab offers a substantial LDL-C reduction, filling a therapeutic gap for HoFH patients, who do not adequately respond to conventional therapies.

The long-term effects of evinacumab in HoFH remain to be fully characterized. This study aims to provide new safety and efficacy data obtained in clinical practice. This trial offers valuable insights into the impact of this therapy, addressing an important gap in long-term treatment outcomes for this patient population.

## Methods

### Patients’ Characteristics

This study included a cohort of 7 patients (3 men and 4 women) aged between 27 and 43 years affected by HoFH by genetic criteria from our Department of Endocrinology and Metabolism (Table 1). Two patients carried *LDLRAP1*-related pathogenic variants (biallelic recessive, monogenic), both with 2 copies of the same variant. The remaining 5 patients had *LDLR*-related, monogenic pathogenic variants: 2 carried one copy of the same variant, while the other 3 each carried one copy of 2 different variants (the genotypes of the subjects are shown in Table 2). One patient additionally had hyperlipoproteinemia(a). No relevant clinical differences in LDL-C values or comorbidities profiles were observed between these 2 groups.

**Table 1:** Patient Characteristics, Including Age in Years, Cardiovascular Comorbidities, and Lipid-Lowering Therapy

Subject	Age	Comorbidities	Lipid-lowering therapy	Apheresis
1	31	Carotid plaques	Rosuvastatin 40 mg, Ezetimibe 5 mg	-
2	40	Carotid plaques	Atorvastatin 20 mg, Ezetimibe 10 mg, Bempedoic Acid 90 mg, Alirocumab 150 mg	-
3	42	PAD, CAD, Carotid plaques	Atorvastatin 80 mg, Ezetimibe 10 mg, Evolocumab 140 mg	Weekly
4	27	-	Atorvastatin 40 mg, Ezetimibe 10 mg, Evolocumab 140 mg	Weekly
5	42	Carotid plaques	Atorvastatin 80 mg, Ezetimibe 10 mg, Bempedoic Acid 180 mg, Inclisiran 284 mg	-
6	43	CAD, Carotid stenosis	Atorvastatin 10 mg, Ezetimibe 10 mg, Bempedoic Acid 180 mg, Alirocumab 150 mg	Weekly
7	41	Carotid plaques	Atorvastatin 80 mg, Ezetimibe 10 mg	Weekly

Abbreviations: CAD, coronary artery disease; PAD, peripheral artery disease.

**Table 2:** Pathogenic Genotypes Identified in Our Cohort

Subj.	Sex	FH diagnosis	Pathogenic variants
1	M	Biallelic recessive hypercholesterolaemia: monogenic, *LDLRAP1*-related (2 copies of the identical variant)	c.649G>T, p.Glu217Ter
2	F	Biallelic semi-dominant hypercholesterolaemia: monogenic, *LDLR*-related (2 copies of the identical variant)	c.1618G>A, p.Ala540Thr
3	F	Biallelic semi-dominant hypercholesterolaemia: monogenic, *LDLR*-related (2 copies of the identical variant)	c.259T>C, p.Trp87Arg
4	F	Biallelic semi-dominant hypercholesterolaemia: monogenic, *LDLR*-related (1 copy each of 2 different variants)	c.986G>A, p.Cys329Tyr c.2054C>T, p.Pro685Leu
5	F	Biallelic recessive hypercholesterolaemia: monogenic, *LDLRAP1*-related (2 copies of the identical variant)	c.606dupC, p.Lys204Glufs∗17
6	M	Biallelic semi-dominant hypercholesterolaemia: monogenic, *LDLR*-related (1 copy each of 2 different variants)	c.796G>A, p.Asp266Asn c.1775G>A, p.Gly592Glu
7	M	Biallelic semi-dominant hypercholesterolaemia: monogenic, *LDLR*-related (1 copy each of 2 different variants)	c.169G>A, p.Asp57Asn c.798T>A, p.Asp266Glu

Abbreviation: LDLR, LDL receptor.

Two patients carried LDLRAP1-related variants (biallelic recessive, monogenic), both with 2 copies of the same variant, the remaining 5 patients had LDLR-related, semi-dominant, monogenic variants: 2 carried one copy of the same variant, while the other 3 each carried one copy each of 2 different variants.

The subjects had varying cardiovascular comorbid conditions. Four subjects had carotid plaques (Subjects 1, 2, 3, 5 and 7), one had carotid stenosis (Subject 6), one had coronary artery disease (CAD) and subject 3 had both peripheral artery disease and CAD. Only our youngest patient (Subject 4) had no cardiovascular comorbidities. Of the 2 subjects presenting with CAD, one was treated at the age of 33 first with a coronary artery bypass graft and later with a percutaneous transluminal angioplasty with stent implantation. Regarding the second subject, the CAD was first diagnosed at the age of 31 and treated with percutaneous transluminal angioplasty with stent implantation, with the procedure repeated 2 years later because of a progression of the 3-vessel disease. Moreover, this subject was also affected by hyperlipoprotein(a). More detailed patient characteristics are summarized in Table 1 and the pathogenic genetic variants are listed in Table 2.

All subjects received the maximum tolerated LLT, including lipid apheresis for 4 of them.

### Study Design

Each patient received evinacumab at a dose of 15 mg per kg of body weight intravenously every 4 weeks, except for subject 1 who received it at longer intervals (6 to 8 weeks) due to logistical issues. The data for this last subject were analyzed separately and not included in the calculation of the mean values.

In one patient, therapy had to be discontinued after 3 doses due to a suspected allergic reaction (sensation of swelling in the upper airways and dyspnea, while the vital parameters remained stable).

The subject 7 decided to suspend the treatment after 28 weeks due to logistical and work-related reasons. This decision was taken independently of the tolerability and he did not report any adverse event during the treatment period.

Clinical parameters, side effects and lab assessment, including plasma creatine kinase, plasma aspartate transaminase, alanine transaminase and Gamma-GT, creatinine, total cholesterol, non-HDL-C, LDL-C, and TG, were evaluated every 2 to 8 weeks for all individuals, and weekly for those undergoing apheresis therapy. The effectiveness and safety of the therapy were monitored through these parameters, as well as additional specific measurements as needed.

Since the study was conducted in a real-world setting, the evaluation of the clinical lab parameters was adapted to the clinical indication as well as to logistic and needs of the patients.

### Statistical Analysis

Percent and absolute changes from baseline in LDL-C, along with changes in other lipid and laboratory parameters, were analyzed using descriptive statistics and are reported as mean value ± SD. Changes in continuous variables were assessed with Student’s *t*-test.

## Results

The average LDL-C under LLTs (including lipoprotein apheresis [LA] for subjects 3, 4, 6, and 7) among the participants at baseline was 222.4 ± 78.8 mg/dl, while the mean treatment-naive LDL was 586.7 ± 96.1 mg/dl. Regarding LLT regimens, 2 subjects (subjects 1 and 7) were treated exclusively with high-intensity statin therapy in combination with ezetimibe, resulting in baseline LDL-C levels of 174 mg/dL and 132 mg/dL (LLTs and LA), respectively. Two subjects (subjects 3 and 4) received high-intensity statin therapy, ezetimibe, and PCSK9 inhibitors, with LDL-C levels of 166 mg/dL and 329 mg/dL, respectively.

The remaining 3 subjects were treated at baseline with high-intensity statin therapy, ezetimibe, bempedoic acid, and PCSK9 inhibitors, achieving LDL-C levels of 320 mg/dL, 235 mg/dL, and 193 mg/dL.

Patients who did not receive bempedoic acid or PCSK9 inhibitors were not treated with these agents due to previous intolerance or insufficient LDL-C reduction.

As for the lipid profile, before starting therapy with evinacumab, the following mean values were measured: TGs 97.7 ± 57.9 mg/dL, total cholesterol 288.9 ± 84.3 mg/dL, HDL-C 45.0 ± 10.6 mg/dL (lipid profile data are summarized in Table 3).

**Table 3:** Lipid Assessment at Baseline and Changes Over the 52-Wk Treatment Period

Parameter	Baseline (mg/dL)	Change from baseline (%)	Absolute reduction from baseline (mg/dL)
LDL-C	222.4 ± 78.8	−68.9 ± 14.0	−145.2 ± 67.0
Total cholesterol	288.9 ± 84.3	−62.7 ± 22.7	−170.0 ± 70.1
Triglycerides	97.7 ± 57.9	−39.5 ± 29.8	−55.2 ± 55.8
HDL-C	45 ± 10.6	−38.4% ± 8.6	−16.3 ± 4.4

Abbreviations: HDL-C, high-density lipoprotein cholesterol; LDL-C, low-density lipoprotein cholesterol.

Following the first administration of evinacumab, an average reduction of LDL-C from baseline of −61.6 ± 14.5% was observed among all participants. After the second and third administrations, LDL-C reduction remained stable with −63.0 ± 13.2% and −62.6 ± 13.1%, respectively. Among the treatment period of 52 weeks, an average decrease from baseline of −68.9 ± 14.0% (*P* < 0,001) has been observed (Table 3). Furthermore, in one patient, who opted for longer intervals between administrations, LDL-C mean reductions of −56.0% and −41.2% were achieved after therapy intervals of 6 and 8 weeks, respectively.

**Fig. 1:**
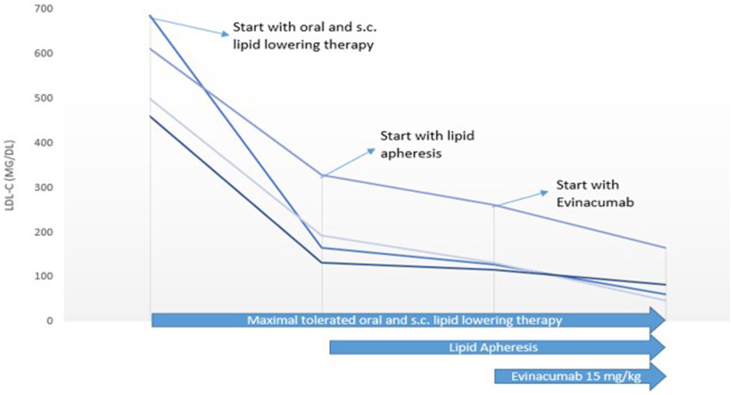
LDL-C reduction over 52 weeks: trends in LDL levels measured every 4 weeks over a 12-month period. Therapy was discontinued after 3 doses due to a suspected allergic reaction in subject 4, and after 28 weeks by subject 7 for logistical reason. Rebound in LDL-C levels observed in patients 2 and 5 due to temporary discontinuation of concomitant oral lipid-lowering therapies. *LDL-C =* low-density lipoprotein cholesterol.

As a result of LDL-C reduction, LA was discontinued in 2 subjects,^3^^,^^7^ and 5 subjects achieved ESC/EAS-recommended LDL-C targets, 3 of them without the need for LA.^1^^,^^2^^,^^5^ Subject 3 subsequently resumed LA after evinacumab was discontinued due to a suspected allergic reaction. Subject 6 continued LA because of elevated lipoprotein(a) levels. The reduction of LDL-C over time across therapeutic escalation in patients treated with LA is shown in Figure 1.

High triglyceride levels are not typically observed in individuals with HoFH. In our cohort, the mean triglyceride level was 97.7 ± 57.9 mg/dL, which falls within the target range according to the 2019 ESC/EAS Guidelines.^24^ Over the 52-week treatment period, a mean reduction of −39.5 ± 29.8% was noted. The observed reductions in triglyceride levels varied considerably across the subjects, which can be attributed to the broad range of values exhibited at baseline (47-223 mg/dL).

Reductions in plasma levels of total cholesterol (−62.7 ± 22.7%) and HDL (−38.4 ± 8.6%) could be observed as well.

### Adverse Events

No side effects were observed except for a suspected allergic reaction, which led to discontinuation of the therapy after the third administration. However, allergic testing did not show any correlation with evinacumab or any of its components. Therefore, re-exposure to the drug is planned under medical supervision and in a monitored setting.

None of the remaining participants experienced any of the typical known side effects of evinacumab (nasopharyngitis, nausea, influenza-like illness, dizziness, extremity pain, rhinorrhea, and injection site reactions).^5^

No cardiovascular events were reported during the 52-week observation period.

Creatine kinase, aspartate transaminase, alanine transaminase, and Gamma-GT serum levels remained stable during the treatment with evinacumab (data not reported).

## Discussion

For the patient population under observation, meeting the LDL-C target as outlined in the 2019 ESC/EAS guidelines proves challenging.^24^

In this study, five of the seven patients achieved the LDL-C level recommended by the guideline. Of the 4 patients undergoing LA, 2 were able to discontinue it, with one patient continuing treatment due to lipoproteinemia(a), while the other had to restart LA after experiencing the suspected allergic reaction to evinacumab. The other 3 subjects that initially refused LA could reach their LDL-C target with evinacumab additionally the standard LLT and without the need to be treated with lipid-apheresis.

The achieved results on lipid parameters showed a slightly greater reduction in LDL compared to the previous studies.^14^^,^^22^^,^^23^ Variable percentages of LDL-C reduction were also observed within our cohort. For example, patient 3 experienced a less pronounced reduction compared to the others. This is likely attributable to differences in the tolerability of other LLTs, resulting in varying baseline LDL-C levels, as well as differences in genotype.

**Fig. 2:**
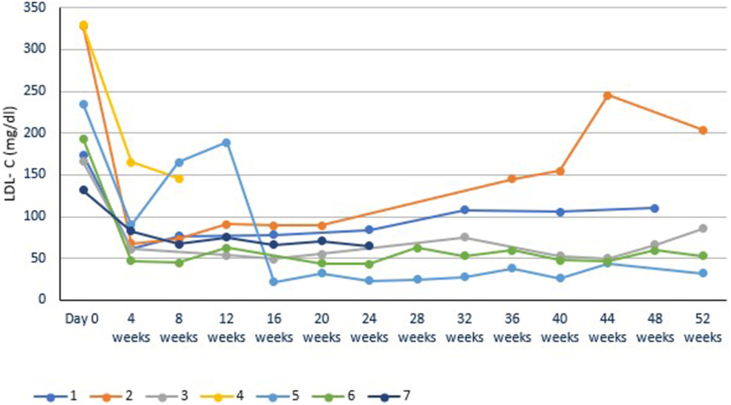
The graphic shows the effects of the progressive escalation of the lipid-lowering therapies in the 4 patients undergoing lipid apheresis (LDL-c values are computed using kroon’s formula),over the years. *LDL-C =* low-density lipoprotein cholesterol.

During the observational period, a rebound in LDL-C levels was observed in 2 subjects (patients 2 and 5, Fig. 2). This is likely attributable to a compliance issue, as both patients had discontinued their oral LLTs, assuming evinacumab and PCSK-9 inhibitors alone would be sufficient. Both subjects were subsequently instructed on the importance of continuing all prescribed LLTs.

Evinacumab appears to be a valuable alternative or adjunctive therapy for patients on LA, helping to reduce time consumption and improving the quality of life of the subjects. Moreover, evinacumab ensures a consistent reduction in LDL-C, unlike the typically fluctuating trends observed with LA.

It is widely recognized that elevated LDL-C levels are associated with an increased cardiovascular risk, which suggests that our cohort with high cardiovascular risk profiles could potentially benefit from this therapy in the long term and we expect to observe an improvement of the long-term prognosis.

The findings suggest that evinacumab is an effective and viable treatment option for patients with HoFH who fail to reach LDL-C targets despite receiving maximal tolerated lipid therapy, including the lipid apheresis.

A decrease in HDL levels was observed as well, which is more pronounced than the reductions observed following the lipid apheresis sessions, which are, as for LDL-C, fluctuating and not constant. However, the clinical relevance of this reduction remains unclear.

### Limitations of the Study

The main limitation of this trial is given by the small sample size. As mentioned above, the achieved results on lipid parameters showed a slightly greater reduction in LDL compared to the previous studies.^14^^,^^22^^,^^23^

The second limitation is given by the real-world clinical context, which implies that the treatment adherence may vary among patients. Even the frequency and timing of laboratory monitoring, as well as drug administrations, have been often influenced by individual circumstances, including patients' availability, willingness to continue the therapy and level of insight into their disease.

## Conclusion

Therapy with evinacumab on top of the best standard of care treatment is an effective way to further reduce LDL-C levels in cardiovascular high-risk patients affected by HoFH.

Our findings suggest that even dosing intervals ranging from 6 to 8 weeks result in a notable reduction in the LDL-C, although not as effective as monthly administration.

Drugs like evinacumab promise to bring a transformation in the therapeutic paradigm for patients affected by HoFH, who, despite requiring life-long therapy, will benefit from a less invasive treatment with a different impact on their daily lives.

## Disclosure

LV has received honoraria from Sobi. DS has received honoraria from Boehringer, MSD, and Novartis. KM has received honoraria from NovoNordisk, Amgen, Sanofi, MSD, and Boehringer. EST has received honoraria from Fresenius Medical Care, Daiichi Sankyo, Amgen, Novartis, Pfizer, Amarin, and Sanofi. UK has received honoraria from Daiichi Sankyo, Amgen, MSD, Sobi, and Synlab. TB has received honoraria from Amgen, Novartis, and Daiichi Sankyo.
